# Supplementary material for: Heparin-based hydrogel scaffolding alters the transcriptomic profile and increases the chemoresistance of MDA-MB-231 triple-negative breast cancer cells
Source: Biomater Sci. 2020 Feb 13;8(10):2786–96. doi: 10.1039/c9bm01481k (PMC7497406; doi:10.1039/c9bm01481k)
Supplement: Supplementary file 2 [file BM-008-C9BM01481K-s002.zip › Supplementary File 4/HP-B hydrogel + EGF (s)vControl/Pathways/my_analysis.Gsea.1545201170987/HALLMARK_APICAL_SURFACE.html]

Details for gene set HALLMARK\_APICAL\_SURFACE[GSEA]

|  || Dataset | expr.class.cls#HYDEGF\_versus\_CONTROL.class.cls#HYDEGF\_versus\_CONTROL\_repos |
| Phenotype | class.cls#HYDEGF\_versus\_CONTROL\_repos |
| Upregulated in class | CONTROL |
| GeneSet | HALLMARK\_APICAL\_SURFACE |
| Enrichment Score (ES) | -0.28525108 |
| Normalized Enrichment Score (NES) | -0.9563355 |
| Nominal p-value | 0.5423341 |
| FDR q-value | 0.5829027 |
| FWER p-Value | 1.0 |
Table: GSEA Results Summary

  

Fig 1: Enrichment plot: HALLMARK\_APICAL\_SURFACE      
 Profile of the Running ES Score & Positions of GeneSet Members on the Rank Ordered List

  

| PROBE | DESCRIPTION (from dataset) | GENE SYMBOL | GENE\_TITLE | RANK IN GENE LIST | RANK METRIC SCORE | RUNNING ES | CORE ENRICHMENT || 1 | SCUBE1 | na |  |  | 200 | 2.607 | 0.0817 | No |
| 2 | MDGA1 | na |  |  | 1421 | 1.459 | 0.0696 | No |
| 3 | ATP6V0A4 | na |  |  | 1468 | 1.443 | 0.1182 | No |
| 4 | SLC34A3 | na |  |  | 2471 | 1.134 | 0.1061 | No |
| 5 | GATA3 | na |  |  | 2562 | 1.112 | 0.1407 | No |
| 6 | CROCC | na |  |  | 3111 | 0.979 | 0.1467 | No |
| 7 | SHROOM2 | na |  |  | 3748 | 0.856 | 0.1437 | No |
| 8 | ATP8B1 | na |  |  | 4436 | 0.744 | 0.1342 | No |
| 9 | EPHB4 | na |  |  | 4726 | 0.702 | 0.1439 | No |
| 10 | AKAP7 | na |  |  | 5615 | 0.559 | 0.1174 | No |
| 11 | AFAP1L2 | na |  |  | 5628 | 0.557 | 0.1364 | No |
| 12 | PCSK9 | na |  |  | 6303 | 0.463 | 0.1176 | No |
| 13 | BRCA1 | na |  |  | 6597 | 0.424 | 0.1173 | No |
| 14 | CD160 | na |  |  | 10267 | -0.018 | -0.0734 | No |
| 15 | NCOA6 | na |  |  | 11388 | -0.145 | -0.1267 | No |
| 16 | PLAUR | na |  |  | 14166 | -0.503 | -0.2538 | No |
| 17 | TMEM8B | na |  |  | 14230 | -0.509 | -0.2391 | No |
| 18 | FLOT2 | na |  |  | 14744 | -0.597 | -0.2448 | No |
| 19 | ADIPOR2 | na |  |  | 15521 | -0.725 | -0.2596 | Yes |
| 20 | CX3CL1 | na |  |  | 15637 | -0.749 | -0.2391 | Yes |
| 21 | LYN | na |  |  | 16413 | -0.926 | -0.2468 | Yes |
| 22 | GSTM3 | na |  |  | 16738 | -1.008 | -0.2281 | Yes |
| 23 | SRPX | na |  |  | 17235 | -1.154 | -0.2132 | Yes |
| 24 | DCBLD2 | na |  |  | 17441 | -1.232 | -0.1803 | Yes |
| 25 | EFNA5 | na |  |  | 17521 | -1.267 | -0.1397 | Yes |
| 26 | HSPB1 | na |  |  | 17580 | -1.287 | -0.0972 | Yes |
| 27 | B4GALT1 | na |  |  | 18015 | -1.493 | -0.0671 | Yes |
| 28 | ADAM10 | na |  |  | 18162 | -1.589 | -0.0185 | Yes |
| 29 | APP | na |  |  | 18704 | -2.052 | 0.0258 | Yes |
Table: GSEA details [plain text format]

  

Fig 2: HALLMARK\_APICAL\_SURFACE      
 Blue-Pink O' Gram in the Space of the Analyzed GeneSet

  

Fig 3: HALLMARK\_APICAL\_SURFACE: Random ES distribution      
 Gene set null distribution of ES for **HALLMARK\_APICAL\_SURFACE**
